# Elucidating the causal relationship between 486 genetically predicted blood metabolites and the risk of gastric cancer: a comprehensive Mendelian randomization analysis

**DOI:** 10.3389/fonc.2024.1418283

**Published:** 2024-12-05

**Authors:** Lei Qian, Jiawei Song, Xiaoqun Zhang, Yihuan Qiao, Zhaobang Tan, Shisen Li, Jun Zhu, Jipeng Li

**Affiliations:** ^1^ Department of Experiment Surgery, Xijing Hospital, Fourth Military Medical University, Xi’an, China; ^2^ School of Clinical Medicine, Xi’an Medical University, Xi’an, China; ^3^ Department of Pharmacy, Shaanxi Provincial Hospital of Chinese Medicine, Xi’an, China; ^4^ Department of Digestive Surgery, Honghui Hospital, Xi’an Jiaotong University, Xi’an, China; ^5^ Department of Gastrointestinal Surgery, Xijing Hospital, Fourth Military Medical University, Xi’an, China; ^6^ Department of Digestive Diseases, Xijing Hospital, Fourth Military Medical University, Xi’an, China

**Keywords:** blood metabolites, gastric cancer, Mendelian randomization, causality, genomewide association study

## Abstract

**Background:**

Previous epidemiological studies have yielded inconclusive results regarding the causality between blood metabolites and the risk of gastric cancer (GC). To address this shortcoming, we conducted a two-sample Mendelian randomization (MR) study, combined with metabolomics techniques, to elucidate the causality between 486 genetically predicted blood metabolites and GC.

**Methods:**

MR analysis and metabolomics techniques such as ultra-high performance liquid chromatography/tandem mass spectrometry (UPLC-MS/MS) and gas chromatography/tandem mass spectrometry (GC-MS/MS) technologies were employed to assess the causality of 486 genetically predicted blood metabolites on the risk of GC. The genome-wide association study (GWAS) summary data for 486 blood metabolites from 7,824 individuals. The GWAS summary data for GC (ebi-a-GCST90018849) were obtained from the IEU Open GWAS project, including 1,029 GC cases and 474,841 controls. Primary causality estimates were obtained using inverse variance weighting (IVW), supplemented with the weighted median, MR-Egger, weighted mode, and simple mode. In addition, we conducted sensitivity analyses (including Cochran’s Q, MR-Egger intercept, MR-PRESSO, and leave-one-out tests),Steiger’s test, linked disequilibrium score regression, and multivariate MR (MVMR) to improve the assessment of causality between GC and blood metabolite. Finally, we recruited a total of 11 patients diagnosed with gastric cancer from the First Affiliated Hospital of Air Force Military Medical University between September and October 2024. The control group comprised 11 healthy individuals. Serum samples were collected from both groups for the evaluation of blood-related metabolite expression levels using advanced techniques such as ultra-performance liquid chromatography-tandem mass spectrometry (UPLC-MS/MS) and gas chromatography-mass spectrometry (GC-MS/MS).

**Results:**

The MVMR analysis revealed a significant association between genetically predicted elevated levels of tryptophan (odds ratio [OR] = 0.523, 95% confidence interval [CI] = 0.313–0.872, p = 0.013), nonadecanoate (19:0) (odds ratio [OR] = 0.460, 95% confidence interval [CI] = 0.225–0.943, p = 0.034), and erythritol (odds ratio [OR] = 0.672, 95% confidence interval [CI] = 0.468–0.930, p = 0.016) with a decreased risk of gastric cancer. Based on metabolomic techniques such as UPLC-MS/MS and GC-MS/MS analyses, it has been demonstrated that the expression levels of tryptophan, nonadecanoate (19:0), and erythritol are reduced in patients with gastric cancer. This finding aligns with the results obtained from our MR analysis and provides further confirmation regarding the protective role of tryptophan, nonadecanoate (19:0), and erythritol against gastric cancer.

**Conclusions:**

These findings indicate that three blood metabolites are causally related to GC and provide new perspectives for combining genomics and metabolomics to study the mechanisms of metabolite-mediated GC development.

## 1 Introduction

Gastric cancer (GC), primarily characterized as an adenocarcinoma, ranks as fifth most prevalent malignancy and the third leading cause of cancer-related mortality globally in 2020 (1). For early-stage GC, endoscopic mucosal dissection is the main therapeutic approach, boasting an impressive 5-year postoperative survival rate of 92.6% (2). For patients with stage I/II GC who undergo laparoscopic or open distal gastrectomy, the survival rate is also commendable but slightly lower, ranging from 73% to 76% (3). However, it’s crucial to note that the overall 5-year survival rate for GC patients, particularly those diagnosed at advanced stages, remains suboptimal, with a median survival of less than one year (4). This disparity underscores the critical need for early detection and intervention to not only enhance survival rates but also significantly reduce healthcare costs. By focusing on prevention and early treatment, we can potentially alleviate the economic burden of GC on both patients and healthcare systems.

Currently, the diagnosis of GC relies primarily on endoscopic and biopsy-based procedures. Although reliable, these methods have drawbacks, such as high financial cost, invasiveness, potential complications, and limited testing resources, which may discourage patient compliance and make them unsuitable for widespread screening initiatives. An ideal alternative would be noninvasive blood tests. Currently used gastrointestinal tumor markers include glycan antigen 199 (CA199) (5) and carcinoembryonic antigen (CEA) (6). Unfortunately, although highly specific, they have low sensitivity and significant rates of false-negative and false-positive results. This calls for further research, possibly in areas such as blood metabolomics, to identify novel biomarkers indicative of GC and facilitate early detection and treatment.

Metabolomics can identify cancer biomarkers and determinants of tumorigenesis by detecting changes in relevant metabolites over the course of disease progression (7). Ikeda et al. found pronounced differences in the serum metabolic profiles of individuals with gastrointestinal malignancies, including esophageal, gastric, and colorectal cancers, compared with those of healthy volunteers (8). Specifically, changes in 3-hydroxypropionic and pyruvic acid levels were found to be sufficiently discriminative to differentiate gastric, esophageal, and colorectal cancers, exceeding the sensitivity and specificity of conventional biomarkers such as CA199 and CEA (8). However, the scientific landscape is currently characterized by a paucity of comprehensive investigations to establish a causal relationship between blood metabolites and GC. Translating these metabolic discoveries into pathophysiological mechanisms and innovative therapeutic strategies remains challenging. Therefore, there is a need for a comprehensive analysis of the interplay between genetic elements and circulating blood metabolites in the etiology of GC.

Mendelian randomization (MR) has emerged as a key methodology in epidemiological research. It derives putative causal relationships between environmental exposure and health outcomes by using distinctive single nucleotide polymorphisms (SNPs) as instrumental variables (9). MR exploits genetic variability to simulate the construct of randomized controlled trials (RCTs). The use of independent genome-wide association study (GWAS) datasets provides the flexibility to independently assess SNPs associated with both exposure and outcome, thereby facilitating two-sample analysis. This technique provides compelling evidence for a causal relationship between disparate phenotypes. By carefully exploring the potential causal relationships between genetic predisposition to disease and various biological traits (e.g., blood metabolomics), MR paves the way for the identification of relevant disease-related biomarkers (10).

Numerous studies have investigated the association between various exposures and GC using magnetic resonance imaging. These investigations have predominantly focused on single exposures or prevalent exposure factors, including body mass index (11), interleukin-6 (12), vitamin D (13), lifestyle patterns such as smoking and alcohol consumption (14), sleep habits (15), and immunoproteins (16). However, investigations of blood metabolites related to GC are scarce. Given the vague nature of the causal link between blood metabolites and GC, we used a two-sample MR methodology, combined with metabolomics techniques, to investigate the causal dynamics between 486 human blood metabolites and GC.

## 2 Materials and methods

### 2.1 Study design

**Figure 1:**
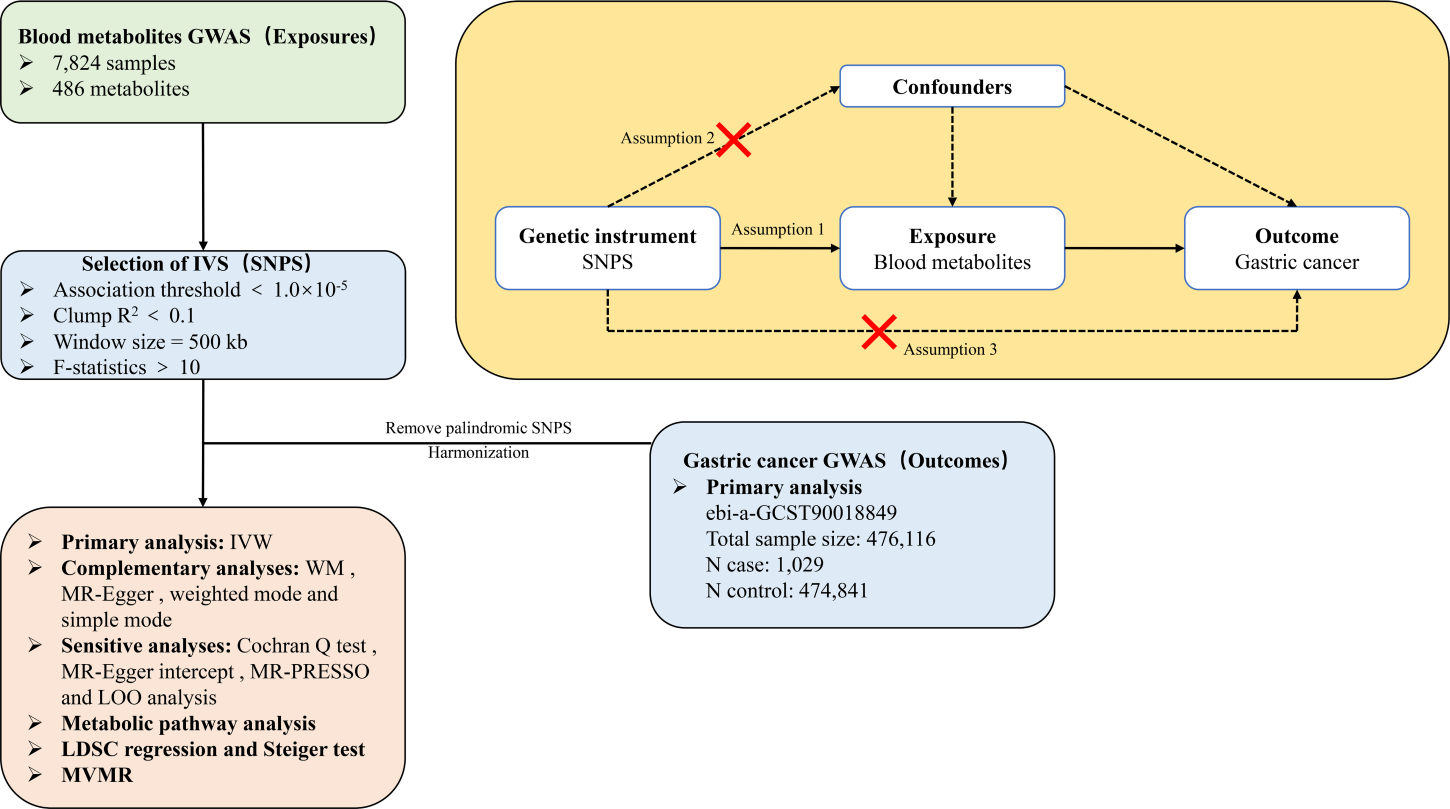
Overview of the research workflow.

In MR research, the integrity of conclusions depends on three key assumptions (**Figure 1**). (1)Correlation assumption:a robust and statistically significant association between the SNP and exposure of interest. (2)Independence assumption: the SNP is free of any correlation with potential confounding variables. (3)Exclusivity assumption: the influence of an SNP on outcomes is exclusively mediated by exposure, ruling out any unaccounted pathways. Based on these principles, our methodology included the selection of high-quality comprehensive datasets from accessible GWASs. This allowed us to obtain appropriate instrumental variables (IVs) for the MR analyses, which were critical for identifying the relationships between an array of 486 blood metabolites and susceptibility to GC.

### 2.2 GWAS data sources

A blood metabolite profiling dataset was obtained from the Comprehensive Metabolomics GWAS repository (https://metabolomics.helmholtz-muenchen.de/gwas/). This dataset comprises a diverse European cohort of 7,824 individuals, including 1,768 participants from the KORA F4 study in Germany and 6,056 from the UK Twin Study (17). Genome-wide association and high-throughput metabolomic studies have revealed approximately 2.1 million SNPs and 486 different metabolites. Of these, 309 metabolites were identified and characterized. The identified metabolites were systematically classified into the following eight categories based on their chemical properties: amino acids, carbohydrates, cofactors, vitamins, energy substrates, lipids, nucleotides, peptides, and xenobiotics. These 486 metabolites are compiled in **Supplementary Table S1**, with the designation “X-” indicating those with yet-to-be-determined chemical properties.

The GWAS data for GC (ebi-a-GCST90018849) were obtained from the IEU Open GWAS Project (https://gwas.mrcieu.ac.uk/). The detailed attributes of the consolidated GC data are described in **Supplementary Table S2**.

### 2.3 Selection of IVs

In accordance with the fundamental tenets of MR analysis, we meticulously delineated a set of criteria for distinguishing the IVs associated with the 486 metabolites. In keeping with the axiom of relevance, our selection protocol strictly adhered to the established genome-wide significance boundary, setting the threshold to a robust p-value (P < 5 × 10-8). Recognizing the subtleties inherent in the genetic underpinnings of certain metabolites, for which only a few SNPs were uncovered, a more permissive threshold was adopted (P < 1 × 10-5) (18, 19).

In a concerted effort to mitigate the confounding intricacies of linkage disequilibrium, a judicious clustering strategy was used, encompassing a swath of 500 kilobase pairs augmented by a correlation coefficient ceiling of 0.01, to isolate SNPs of sovereign genetic locations. In addition, we removed SNPs carrying mismatched alleles or palindromic sequences, which are indicative of genotyping inaccuracies. Simultaneously, we excluded SNPs that had a statistical synergy with the outcome variable or were absent from the outcome cohort, thus preserving the integrity of the assay.

The veracity of each SNP as an IV was judged through the prism of the F statistic (18), given by the following formula, where “ N ” represents the cohort size and “ $R^{2}$ ” is the proportional variance attributed to the SNPs within the exposome profile.


$$F=\frac{R^{2}\times \left(N-2\right)}{1-R^{2}}$$

The calculation of “ $R^{2}$ ” involved the following formula, where “ EAF ” represents the frequency of the effect allele, “ β ” is the regression coefficient explaining the magnitude of the SNP-exposure association, and “ SD ” is the standard deviation.


$$R^{2}=\frac{2\times EAF\times \left(1-EAF\right)\times \beta^{2}}{SD^{2}}$$

To mitigate bias associated with weak instrumental variables, we excluded SNPs with F < 10. Subsequently, we identified and extracted the SNPs for our exposure of interest from the outcome data while excluding those that were significantly related to the outcome (p < 1 × 10−5). The SNPs that survived the rigorous selection process are shown in **Supplementary Table S2** (20).

### 2.4 Univariate MR analysis

In this study, a quintet of MR assays was used, with the predominant analysis using the inverse variance-weighted (IVW) paradigm for its statistical power. This approach synthesizes Wald ratio calculations for each SNP-outcome conjugation, providing a composite causal estimate (21). In addition, causal associations between a compendium of 486 metabolites and GC susceptibility were assessed using odds ratios (ORs) embedded within 95% confidence intervals (CI).

To strengthen the robustness and credibility of our MR conclusions, additional checks were performed using MR-Egger and weighted median (WM) evaluations. An MR-Egger inspection was implemented to detect and correct the putative pleiotropic effects, thereby providing more reliable estimates. The use of the weighted median method yields robust causal inferences, mitigates Type I errors, and enhances the detection of authentic effects even when a preponderance of the input comes from potentially compromised IVs. Harmonization of the WM and MR-Egger results (P < 0.05) with those of the IVW method, both in terms of the trajectory and magnitude of effect, was essential for confirming the validity of these findings.

MR scatter plots were generated to visualize the hypothesized causal relationship between the identified metabolites and GC risk.

### 2.5 Sensitivity analysis

To unravel the intricacies of SNP heterogeneity, we used Cochran’s Q test within the IVW and MR-Egger frameworks. A P-value < 0.05 served as the arbiter of significant heterogeneity. It is worth noting that this statistical test highlights differences in IV effect sizes. In addition, the MR-Egger intercept coupled with the MR-PRESSO analysis tools was used to expose the spectrum of horizontal pleiotropy, with statistical significance determined using a P-value < 0.05 (22). The robustness of our inferential scaffold was tested using careful leave-one-out (LOO) analysis (23). This rigorous technique ensures that the influence of any single SNP does not unduly bias the overarching determination of causality.

### 2.6 Metabolic pathway analysis

To elucidate the underlying biological mechanisms through which prominent blood metabolites influence GC susceptibility, we expanded our analysis to include metabolic pathway exploration. We performed Kyoto Encyclopedia of Genes and Genomes pathway enrichment analysis using MetaboAnalyst (version 5.0; https://www.metaboanalyst.ca/).

### 2.7 Genetic correlation and direction validation

In the context of dissecting genetic correlations between determinants and clinical outcomes, MR estimation could potentially bias the interpretation of causality (24). To circumvent the confounding complications introduced by the coinheritance of significant metabolites and GC risk, we adopted the Linkage Disequilibrium Score Regression (LDSC) methodology. Additionally, we used the Steiger test to assess the potential for reverse causation, which is a critical step in determining whether genetic variants have a stronger association with the determinant than with the consequence (25). Within the confines of the Steiger framework, a P-value of less than 0.05 is statistically significant, supporting the primacy of genetic instruments in modulating the determinant, thus strengthening our primary hypothesis.

### 2.8 Multivariate Mendelian randomization analysis

Given the interrelationships among the salient metabolites that emerged as statistically significant, we conducted a series of multivariate MR (MVMR) analyses to elucidate the distinct causal contributions of multiple metabolite exposure to GC risk. Our baseline MVMR analysis used an IVW strategy, and we refined our investigation using the MR-PRESSO method to identify and correct for potential genetic-level heterogeneity and the confounding effects of outliers. This meticulous approach refines precision and strengthens the integrity of causal inferences.

### 2.9 Statistical analysis

Each MR study used the “TwoSampleMR” (version 0.4.22) software package for R (version 4.1.2) as the computational framework. LDSC was performed using LDSC software (version 1.0.1). The criterion for statistical significance was set at P < 0.05. The magnitude and direction of the causal associations between variables were quantified using ORs and their respective 95% CIs.

### 2.10 Metabolomic analysis

From September to October 2024, 11 preoperative blood samples from GC patients admitted to the First Affiliated Hospital of Air Force Military Medical University and 11 blood samples from healthy controls were selected for analysis. All samples were stored at -80°C. This study was approved by the Ethics Committee of the First Affiliated Hospital of Air Force Military Medical University (approval number KY20222083-F-1).

Subsequently, an untargeted metabolomic analysis was conducted utilizing ultra-high performance liquid chromatography/tandem mass spectrometry (UPLC-MS/MS) and gas chromatography/tandem mass spectrometry (GC-MS/MS) technologies based on the HD4 high-resolution accurate mass analysis platform. The analysis focused on baseline serum metabolites, including tryptophan, nonadecanoate (19:0), and erythritol. All metabolite data were transformed using a logarithm and normalized on a batch basis. For further details regarding the specific metabolomic analysis methodology, please refer to reference (1, 26).

A comprehensive quality control and management system was implemented throughout the experiment to ensure accurate and consistent identification of the true chemical components and to eliminate any potential interference due to misattribution, background noise or system artifacts. The stability of the instrument’s performance was evaluated by calculating the relative standard deviation (RSD) of the internal standards introduced to each sample prior to injection into the mass spectrometer.

## 3 Results

### 3.1 IVs for exposures

After an exhaustive and methodological selection process, 486 serum metabolites were selected for evaluation using MR analysis. The number of SNPs associated with these metabolites ranged from 3 to 503. Metabolites with the identifiers #00577, #32322, #33188, #34453, and #37459 were distinguished using the sparsest array of genetic tools, each of which was underpinned by only three correlated SNPs. Conversely, metabolite #33178 was located at the apex, with a substantial endowment of 496 SNPs, conferring genetic instrumentation. The F-statistics for all SNPs involved in the correlation analyses uniformly exceeded the threshold of 10, heralding the robust statistical power of the selected IVS and mitigating the risk of bias that could come from weak instruments. For a more detailed view of the IV data, please see **Supplementary Table S2**.

### 3.2 Primary analysis

**Figure 2:**
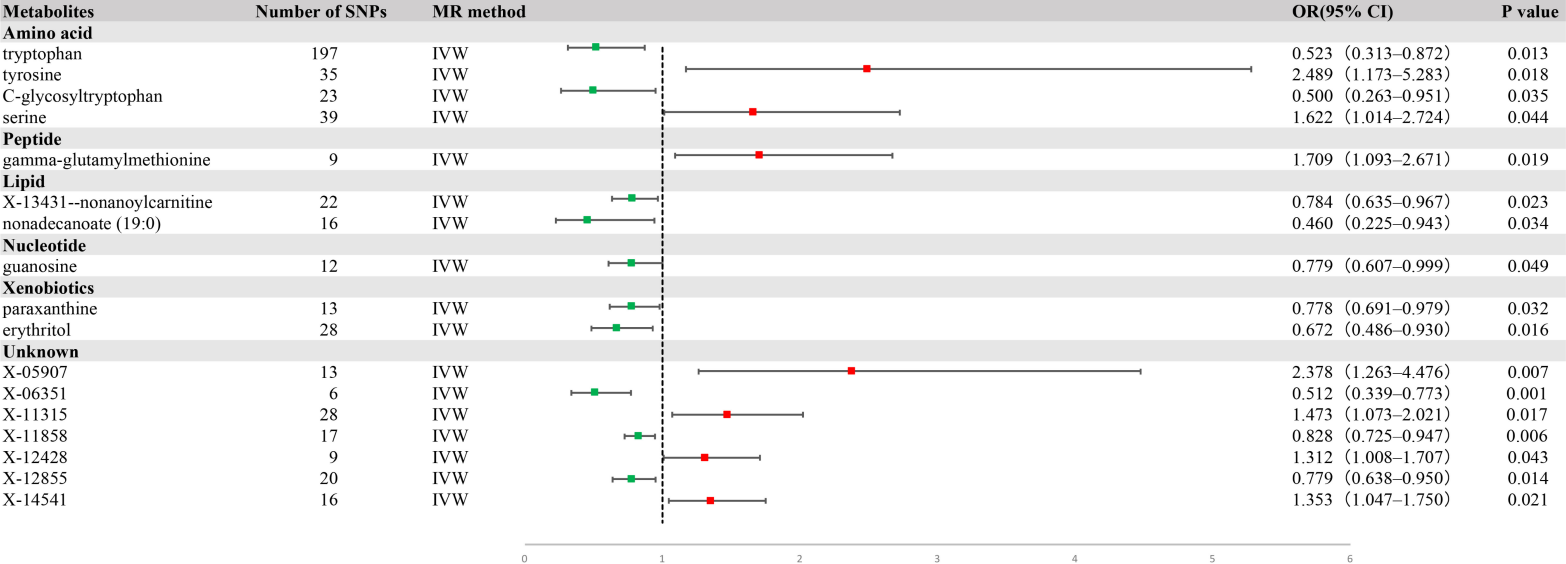
A forest plot of the causal effect of blood metabolites on gastric cancer (GC) risk from univariate Mendelian randomization with inverse variance weighting (IVW).

**Figure 3:**
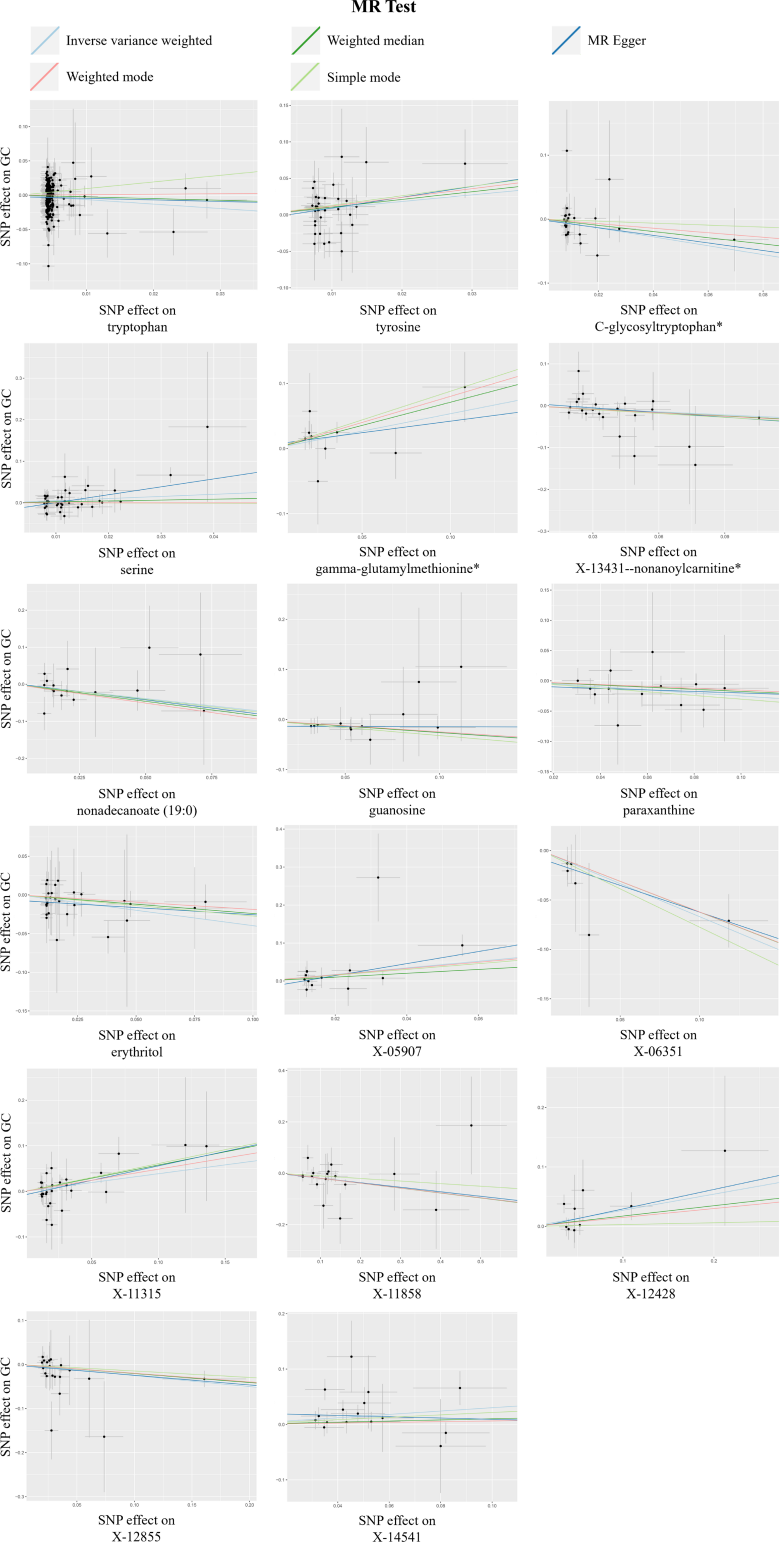
A scatterplot of the significant causal relationship (P < 0.05) between blood metabolites and gastric cancer (GC).

Using IVW analysis, we identified 17 metabolites with potential relevance to GC etiology. Of these, 10 were characterized, whereas the remaining seven were not. These 17 metabolites had compelling associations with susceptibility to GC (**Figure 2**), and spanned a diverse spectrum of chemical classifications, such as amino acids, peptides, lipids, nucleotides, and xenobiotics, including tryptophan (OR = 0.523, 95% CI = 0.313−0.872, P = 0.013), tyrosine (OR = 2.489, 95% CI = 1.173−5.283, P = 0.018), C-glycosyltryptophan (OR = 0. 500, 95% CI = 0.263−0.951, P = 0.035), serine (OR = 1.622, 95% CI = 1.014−2.724, P = 0.044), gamma-glutamylmethionine (OR = 1.709, 95% CI = 1.093−2.671, P = 0. 019), X-13431-nonanoylcarnitine (OR = 0.784, 95% CI = 0.635−0.967, P = 0.023), nonadecanoate (19:0) (OR = 0.460, 95% CI = 0.225−0.943, P = 0.034), guanosine (OR = 0. 779, 95% CI = 0.607−0.999, P = 0.049), paraxanthine (OR = 0.778, 95% CI = 0.691−0.979, P = 0.032), and erythritol (OR = 0.672, 95% CI = 0.486−0.930, P = 0.016) (**Figure 3**).

Within the framework of the IVW method, the concordance of the results derived from the MR-Egger method and weighted median estimations underscored the robustness of the associations between these metabolites and GC risk (**Table 1**).

**Table 1:** Detection of causal relationships between 17 blood metabolites and GC risk using two MR models and tests for heterogeneity and horizontal pleiotropy.

Metabolites	Number of SNPs	MR analysis			Heterogeneity		Pleiotropy	
		Method	OR (95% CI)	P-value	Q	P	Intercept	p
Amino acid								
Tryptophan	197	ME	0.798 (0.211– 3.020)	0.740	211.685	0.196	−0.00234672261246323	0.501
		WM	0.781 (0.320– 1.907)	0.587				
Tyrosine	35	ME	4.419 (0.214–91.054)	0.343	36.552	0.307	−0.00562161200575098	0.703
		WM	2.872 (0.934– 8.837)	0.066				
C-glycosyltryptophan*	23	ME	0.553 (0.165–1.851)	0.347	15.287	0.808	−0.00158207855094606	0.850
		WM	0.616 (0.252–1.508)	0.289				
Serine	39	ME	6.728 (1.889–23.967)	0.006	0.850	0.857	−0.0187715047558943	0.025
		WM	1.241 (0.560– 2.747)	0.595				
Peptide								
Gamma-glutamylmethionine*	9	ME	1.426 (0.550–3.699)	0.489	4.973	0.663	0.007	0.687
		WM	2.037 (1.137–3.651)	0.017				
Lipid								
X-13431–nonanoylcarnitine*	22	ME	0.689 (0.447–1.062)	0.107	16.186	0.705	0.006	0.513
		WM	0.762 (0.569–1.021)	0.068				
nonadecanoate (19:0)	16	ME	0.412 (0.060–2.847)	0.384	10.669	0.712	0.002	0.905
		WM	0.397 (0.142–1.108)	0.078				
Nucleotide								
Guanosine	12	ME	0.989 (0.498–1.964)	0.975	1.527	0.999	−0.0132003086022429	0.481
		WM	0.770 (0.562–1.057)	0.106				
Xenobiotics								
Paraxanthine	13	ME	0.883 (0.440–1.768)	0.731	4.723	0.944	−0.007517064025338	0.714
		WM	0.839 (0.610–1.154)	0.281				
Erythritol	28	ME	0.832 (0.491–1.409)	0.500	11.851	0.992	−0.00721226588820352	0.323
		WM	0.786 (0.496–1.246)	0.306				
Unknown								
X-05907	13	ME	4.891 (1.487–16.090)	0.024	12.980	0.295	−0.0176623721801645	0.194
		WM	1.662 (0.699– 3.951)	0.250				
X-06351	6	ME	0.583 (0.334–1.017)	0.130	0.943	0.918	−0.00823932365242328	0.539
		WM	0.537 (0.303–0.951)	0.033				
X-11315	28	ME	1.886 (1.059–3.359)	0.041	22.996	0.633	−0.00754445825407327	0.325
		WM	1.782 (1.119–2.839)	0.015				
X-11858	17	ME	0.839 (0.607–1.160)	0.305	13.384	0.573	−0.00150060089220439	0.932
		WM	0.826 (0.687–0.992)	0.041				
X-12428	9	ME	1.388 (0.744–2.590)	0.337	6.630	0.468	−0.00338631534292095	0.850
		WM	1.190 (0.824–1.719)	0.353				
X-12855	20	ME	0.803 (0.606–1.063)	0.143	13.474	0.763	−0.00233762913162488	0.768
		WM	0.816 (0.624–1.067)	0.137				
X-14541	16	ME	0.889 (0.405–1.952)	0.774	19.105	0.161	0.021	0.287
		WM	1.108 (0.802–1.531)	0.535				

GC, gastric cancer; ME, MR-Egger.

### 3.3 Sensitivity analysis

To substantiate the robustness of our findings, we conducted a comprehensive series of sensitivity analyses, including Cochran’s Q test, MR-Egger intercept test, MR-PRESSO, and LOO analysis. Cochran’s Q test showed no significant heterogeneity, confirming the uniformity of the dataset. In addition, the MR-Egger intercept test showed no statistical evidence of horizontal pleiotropy (**Table 1**).

**Figure 4:**
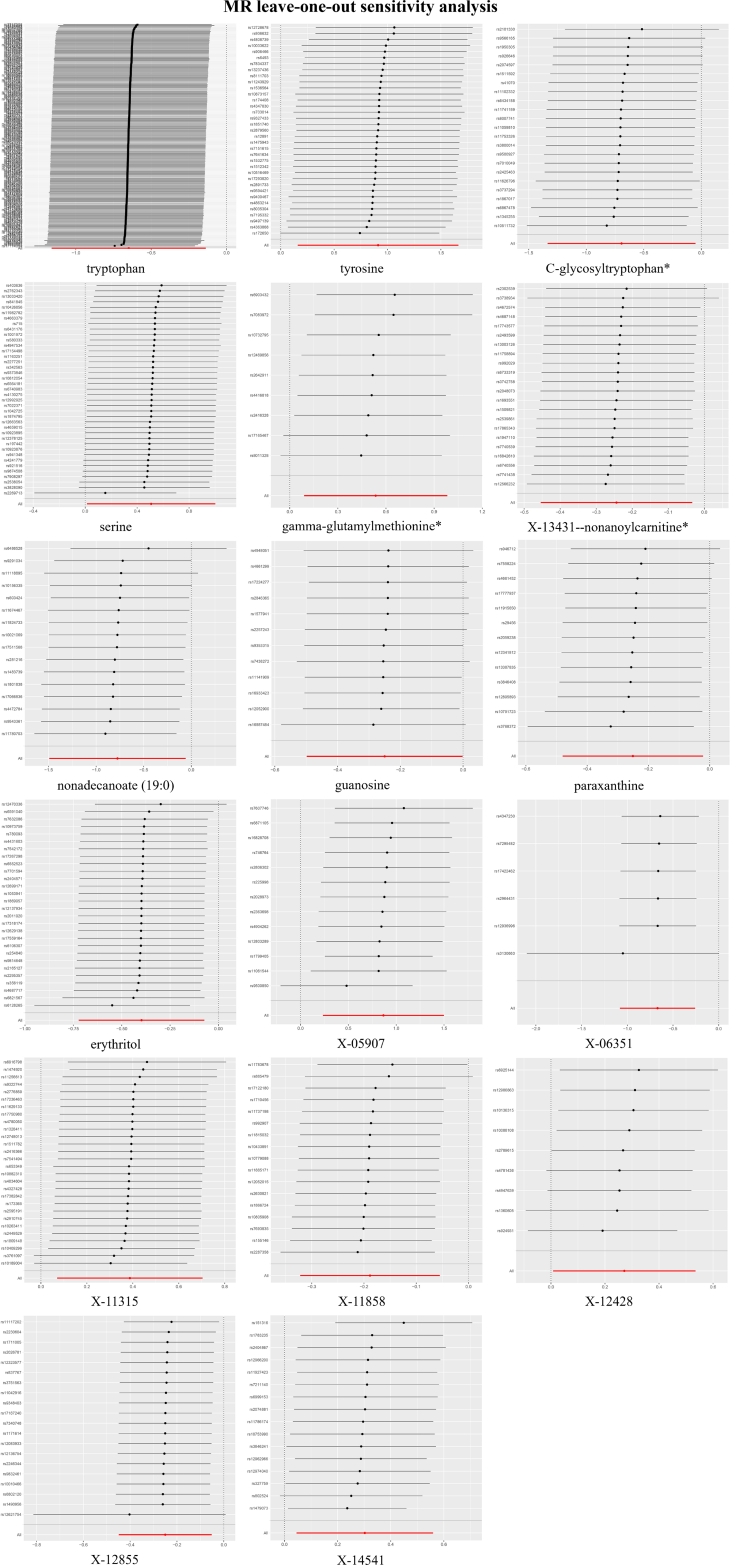
Leave-one-out plots for the causal association between blood metabolites and gastric cancer (GC).

In the LOO analysis, the systematic exclusion and subsequent recalculation of MR estimates for each SNP in isolation confirmed the stability of our findings, indicating that no SNP introduced a consequential bias (**Figure 4**). The MR-PRESSO test, a sentinel test for outlier SNPs that potentially induce heterogeneity, did not reveal any significant differences (**Supplementary Table S3**).

### 3.4 Metabolic pathway analysis

Using insights from the 10 established metabolites, we uncovered a quintet of metabolic pathways potentially integral to GC (**Table 2**): aminoacyl-tRNA biosynthesis; phenylalanine, tyrosine, and tryptophan biosynthesis; ubiquinone and other terpenoid quinone biosynthesis; phenylalanine metabolism; and caffeine metabolism. These pathways may provide the foundation for the biological edifice within which GC emerges.

**Table 2:** Significant metabolic pathways involved in the pathogenesis of gastric cancer (GC).

Metabolic pathways	Involved metabolites	P-value
Aminoacyl-tRNA biosynthesis	L-Tryptophan/L-Tyrosine	0.005417
Phenylalanine, tyrosine and tryptophan biosynthesis	L-Tyrosine	0.010293
Ubiquinone and other terpenoid-quinone biosynthesis	L-Tyrosine	0.023046
Phenylalanine metabolism	L-Tyrosine	0.025582
Caffeine metabolism	1,7-Dimethylxanthine	0.025582

Notably, L-tyrosine was a recurrent participant in the first four enumerated pathways, L-tryptophan was critical to the aminoacyl-tRNA biosynthesis pathway, and 1,7-dimethylxanthine was exclusive to the caffeine metabolism pathway. These results suggest a potential direct involvement in malignant transformation processes that characterize gastric carcinogenesis and invite more exhaustive investigative efforts.

### 3.5 Evaluation of genetic correlation and directionality

Our results indicated a lack of statistically significant genetic correlation, underscoring the elusive nature of the genetic underpinnings that may link these metabolites to GC. Specifically, the regression coefficients (Rg) for tryptophan, tyrosine, guanosine, serine, nonadecanoate (19:0), and X-13431-nonanoylcarnitine were −0.0728, −0.0399, −0.3576, 0. 0674, −0.0276, and −0.1312, respectively, paired with the standard errors (Se) that underscore the imprecision of these estimates (0.0814, 0.1810, 0.2496, 0.2646, 0.1689, and 0.2297, respectively). None reached statistical significance, with p-values exceeding 0.05 (0.3709, 0.8255, 0.1518, 0.7988, 0.8701, and 0.5680, respectively). This finding suggests that the current cohort size was insufficient to detect a clear genetic association. The SNP heritability estimates for these metabolites ranged from 0.0725 (serine) to 0.9757 (tryptophan) (**Supplementary Table S4**).

We also applied the Steiger test to the cohort of 10 recognized metabolites to identify potential reverse causal vectors. Substantial results from the Steiger test rejected the hypothesis of an inverse effect, whereby GC perturbed the levels of these circulating metabolites. The available evidence (**Supplementary Table S5**) does not lend credence to such inverse dynamics.

### 3.6 MVMR analysis

**Figure 5:**
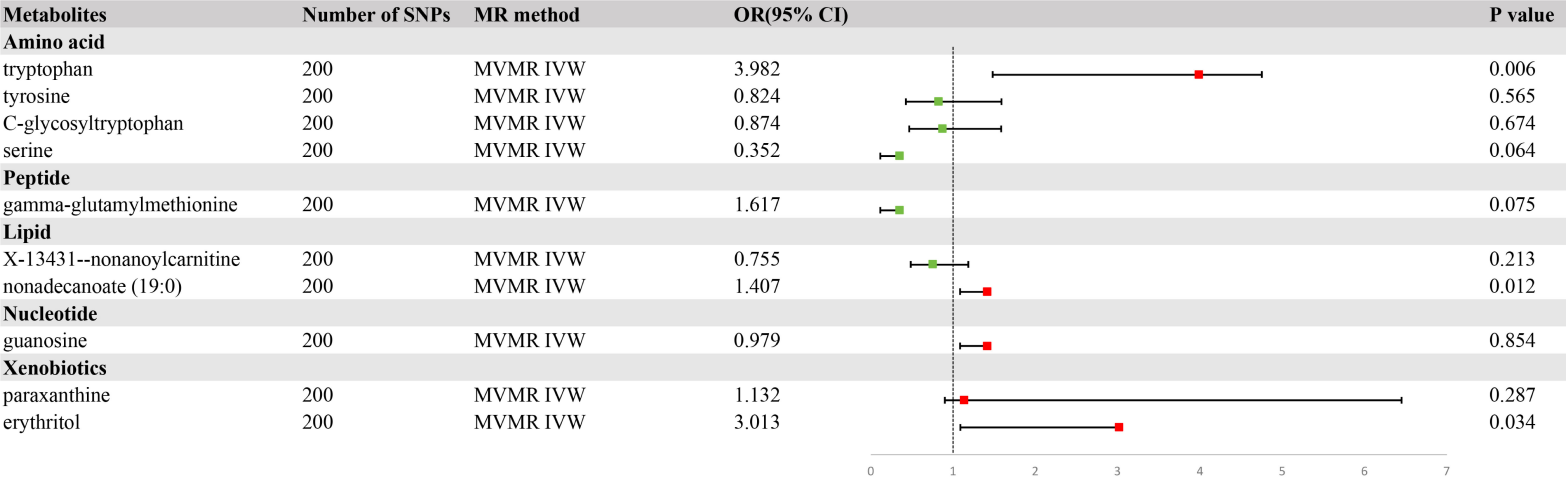
A forest plot of the causal effects of blood metabolites on gastric cancer (GC) risk from multivariate Mendelian randomization with inverse variance weighting (IVW).

To delineate the potential causal relationship between the selected metabolites and GC incidence, we performed MVMR analysis using the IVW method. Simultaneously, we screened the indicators of genetic instrument heterogeneity using the MR-PRESSO approach. Converging evidence from both the IVW and MR-PRESSO analyses suggested that the genetic proxies for tryptophan, nonadecanoate (19:0), and erythritol harbored direct and independent causal links to GC susceptibility, devoid of the confounding effects of other metabolites considered in our investigation (**Figure 5**, **Supplementary Table S6**).

### 3.7 The relative content of tryptophan, nonadecanoate (19:0) and erythritol in human blood samples

**Figure 6:**
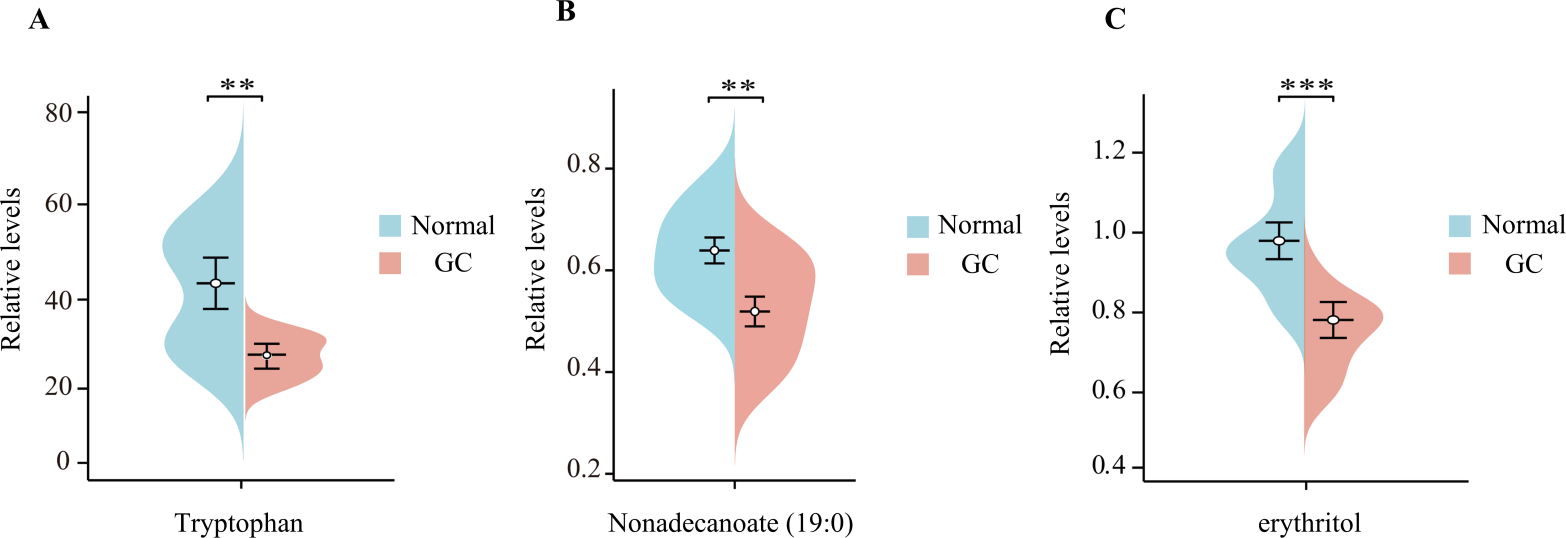
The beanplots of the relative amounts of tryptophan **(A)**, nonadecanoate (19:0) **(B)**, and erythritol **(C)** in blood samples from patients with gastric cancer (GC) compared to those from healthy controls ‘*’ means p < 0.05; ‘**’ means p < 0.01; ‘***’ means p < 0.001.

The relative content of tryptophan, nonadecanoate (19:0) and erythritol was determined by untargeted metabolomics analysis of blood samples collected from hospitals (**Figure 6**). The results demonstrated that the levels of these three substances in the blood of GC patients were markedly diminished in comparison to those observed in healthy control groups (p < 0.05). This finding is consistent with the results of our MR analysis, which provides further confirmation of the role of tryptophan, nonadecanoate (19:0) and erythritol as protective factors against GC.

## 4 Discussion

Based on primary analyses utilizing Inverse Variance Weighting (IVW), weighted median approaches, and MR-Egger regression, along with sensitivity analysis, we identified 17 metabolites that are causally associated with gastric cancer (GC). Among these, 10 are well-documented, including tryptophan, tyrosine, C-glycosyltryptophan, serine, gamma-glutamylmethionine, X-13431-nonanoylcarnitine, nonadecanoate (19:0), guanosine, paraxanthine, and erythritol. Notably, After adjusting for relevant covariates using multivariate MR analysis, the associations of tryptophan, nonadecanoate (19:0), and erythritol with GC risk remained significant. Furthermore, the results of UPLC-MS/MS andvGC-MS/MS showed that compared with the healthy control group, the blood content of these three substances in GC patients was significantly reduced (p < 0.05). This finding is consistent with our MR Analysis and further confirms the role of tryptophan, palmitate (19:0) and erythritol as protective factors for GC. To our knowledge, this is the inaugural MR study to systematically investigate the prospective causal interactions between circulating metabolites and GC risk, highlighting the potential of these metabolites as biomarkers for both screening and therapeutic intervention.

The conclusions drawn from our rigorous MVMR analysis suggested that increased tryptophan, nonadecanoate (19:0), and erythritol levels were associated with a decreased risk of GC progression. This suggestion is supported by literature that implicates tryptophan metabolism as a potential antagonist in oncogenesis. Some tryptophan derivatives are implicated in orchestrating immune responses and limiting neoplastic proliferation. One example is the reduction of glutathione peroxidase 2, which triggers an increase in kynurenine, a tryptophan byproduct. This increase leads to the accumulation of reactive oxygen species via the tryptophan metabolic pathway, impeding the progression and metastatic potential of gastric malignancies (27). Nevertheless, the influence of tryptophan metabolism on carcinogenesis is not unambiguous, with bifurcated pathways toward either oncogenic or tumor-suppressive roles that depend on many elements, including distinct metabolic trajectories, neoplastic typology, tumor microenvironment, and host immune constitution (28).

Nonadecanoate (19:0), a long-chain fatty acid ester, was recently identified as the predominant constituent of essential oil derived from the fruits of Pistacia terebinthus, which exhibits promising antineoplastic properties against lung carcinoma cell lines (29). Unfortunately, the literature on the mechanistic insights related to this monomeric component is scarce.

Erythritol, a tetrahydric alcohol sugar synthesized endogenously from glucose via the pentose phosphate pathway in human cells, is available through dietary channels as a synthetic sweetener. Research has shown that erythritol may exert a critical influence on cerebral oncogenesis and the modulation of hydrogen peroxide, with its action depending on its concentration in biological systems (30).

We also identified a cadre of established metabolites that provide protection against gastric carcinoma. In particular, C-glycosyltryptophan, a metabolite within tryptophan metabolism, has historically been used as an index of renal function (31, 32), with empirical associations suggesting an increase in the infectious and inflammatory burden (33). Its role against GC is supported by correlations with cardiovascular and thyroid pathologies (34, 35). X-13431-nonanoylcarnitine represents a research gap, and its functional parity with recognized acylcarnitines is based on its structural cognate. The physiological and pathophysiological implications of acylcarnitines are manifold, such as influencing the sequelae of myocardial ischemia, glucose homeostasis, and inflammatory processes (36).

In this study, we found an association between elevated levels of tyrosine, serine, and gamma-glutamylmethionine in blood metabolites and an increased risk of GC progression. Tyrosine is involved in gluconeogenesis and ketogenesis, linking energy, lipid, and glucose metabolism. Disorders of tyrosine metabolism have been identified as biomarkers for hepatocellular carcinoma and gastroesophageal malignancies, and alterations in metabolism and related pathways play a key role in cancer development and progression (37, 38). Serine, which is essential for the rapid growth of tumor cells, contributes to the proliferation of colon cancer by providing single-carbon units. In addition, there is evidence that glycine supplementation may alter serine metabolism in tumor cells and that serine deprivation may inhibit tumor growth by affecting lipid metabolism pathways, particularly those involving palmitoyltransferases (39–41). Moreover, a negative correlation between plasma gamma-glutamylmethionine levels and the risk of lethal prostate cancer progression has been observed (42).

Our study has several strengths. First, it adopted an innovative approach by integrating metabolomics and genomics, which differs from previous MR analyses that focused only on single or conventional exposure factors. Moreover, by employing rigorous MR analysis and Steiger’s analysis, we effectively addressed inherent limitations of traditional observational studies, such as reverse causality and confounding biases. Finally, we validated specific metabolite levels in the blood of patients with gastric cancer using metabolomics techniques, further supporting the reliability of the results obtained by Mendelian analysis.

Nevertheless, our study has certain limitations. First, there were a limited number of available SNPs for the exposures at the genome-wide level. To counteract this, we made a deliberate decision to moderately adjust the p-value thresholds in our MR analysis. Nonetheless, it is crucial to emphasize that the F-statistic value for all SNPs we selected surpassed 10, which is a reassuring indicator of the strength and reliability of our instrumental variables. Second, our study did not account for potential confounding factors known to influence gastric cancer incidence, such as smoking and alcohol consumption. Nevertheless, the intercept test from the MR-Egger method yielded P-values above 0.05, indicating that the SNPs associated with the metabolites we selected are not pleiotropic. In other words, these SNPs are unlikely to influence outcomes through pathways unrelated to the metabolites of interest. Despite this, we recognize the importance of considering these confounding factors in future research to gain a more comprehensive understanding of the causal links between blood metabolites and gastric cancer development. Finally,our analysis identified several metabolites as potential risk predictors for GC; however, these metabolites remained uncharacterized. Detailed studies on their molecular structures and functions may reveal novel biomarkers or therapeutic targets, thereby advancing the field of GC research.

In conclusion, our investigation sheds light on the potential causal associations between 10 known metabolites and GC through primary analysis. In addition, MVMR analysis andvmetabolomics techniques suggested that 3 metabolites affect the progression of GC. Our findings highlight the importance of GC in mediating the interplay between metabolites and GC, thereby opening new avenues for research on the etiology of GC, particularly its intersection with environmental factors.

## Data Availability

The original contributions presented in the study are included in the article/**Supplementary Material**. Further inquiries can be directed to the corresponding author.
